# Reliable Positioning and mmWave Communication via Multi-Point Connectivity †

**DOI:** 10.3390/s18114001

**Published:** 2018-11-16

**Authors:** Dileep Kumar, Jani Saloranta, Jarkko Kaleva, Giuseppe Destino, Antti Tölli

**Affiliations:** 1 Centre for Wireless Communications, University of Oulu, 90014 Oulu, Finland; jani.saloranta@oulu.fi (J.S.); jarkko.kaleva@oulu.fi (J.K.); giuseppe.destino@oulu.fi (G.D.); antti.tolli@oulu.fi (A.T.); 2 Centre for Telecommunication Research, King’s College London, London WC2 R2LF, UK

**Keywords:** 5G positioning, coordinated multi-point, mmWave MIMO, beamforming, blockage

## Abstract

One of the key elements of future 5G and beyond mobile technology is millimeter-wave (mmWave) communications, which is targeted to extreme high-data rate services. Furthermore, combining the possibility of a wideband signal transmission with the capability of pencil-beamforming, mmWave technology is key for accurate cellular-based positioning. However, it is also well-known that at the mmWave frequency band the radio channel is very sensitive to line-of-sight blockages giving rise to unstable connectivity and inefficient communication. In this paper, we tackle the blockage problem and propose a solution to increase the communication reliability by means of a coordinated multi-point reception. We also investigate the advantage of this solution in terms of positioning quality. More specifically, we describe a robust hybrid analog–digital receive beamforming strategy to combat the unavailability of dominant links. Numerical examples are provided to validate the efficiency of our proposed method.

## 1. Introduction

To tackle the exponential increase in data traffic, use of millimeter-wave (mmWave) spectrum (30–300 GHz) is gaining more popularity. In fact, mmWave communication is expected to be one of the key components for future 5G system [1]. Ultra-wide band transmission and massive multiple-input–multiple-output (MIMO) techniques at the mmWave frequencies not only contribute in achieving giga-bit wireless access, but also provide accurate positioning capabilities because of higher temporal and angular resolution [2]. Thus, unlike the legacy communication systems, positioning will be an integral feature of mmWave based radio-access-technology (RAT), for example, mentioned in a recent 3GPP study [3].

Widely adopted positioning methods rely mostly on conventional techniques, such as on time(-difference)-of-arrival (ToA/TDoA), angle-of-arrival (AoA) and received-signal-strength (RSS). The key idea is based on estimating the positioning parameters, such as distance, angle or both from two or more base stations (BSs) [2,4]. Related work showed that massive MIMO with full-digital beamforming architecture can provide sub-meter level localization accuracy for both sub-6GHz frequencies [5] and mmWave frequencies [6]. However, full digital solutions are costly and power inefficient when the number of antennas become large due to high power consumption by ADC/DAC and other RF components. Thus, alternative approaches based on all-analog or hybrid structures are needed in practice. Commercially available mmWave hardware features phased-array of antennas with limited number of baseband processing chains [7]. Existing work with such hardware constraints is limited. For example, author of [8] considered Wi-Fi access-points (APs) and localized users with three or more APs, using the conventional triangulation techniques.

In contrast to most prominent localization methods based on signals from two or more BSs, mmWave RAT can accurately estimate user’s position with the single BS by processing orthogonal pilots in different analog beams [9,10]. The author of [9,10] provided closed-form expressions for the Cramér–Rao Lower Bound of the achievable localization accuracy, considering point-to-point communication. Author of [11] studied trade-off between positioning quality and achievable throughput with the increasing training overhead for single-user case and extended to multi-user scenario in [12], while the authors of [13,14] investigated the rate-0positioning trade-off for different beam training strategies. Similar trade-offs are also drawn in [15] for different bandwidths and power splitting factors between positioning and communication.

The mmWave channel is characterized by “quasi”-optical propagation properties [1], thus the main contribution to communications is from the line-of-sight (LoS) path. However, dominant paths are also susceptible to blockage due to relatively low diffraction and higher penetration loss [16], which results in loss of connection and gives rise to unreliable communication. To solve these problems, one possible solution consists of coordinated multi-point reception, whereby enabling each user to have concurrent connections with multiple BSs for better communication robustness [17]. Coordinated transmission/reception has been well studied in the literature over the past decade, mostly under the context of Long Term Evolution (LTE) and LTE-Advanced mobile communication systems [18,19,20]. Widely adapted techniques, such as coordinated beamforming and multi-point selection are included in 3GPP standards [21] and are currently deployed in 4G systems to enhance network capacity and cell-edge performance. However, localization in the conventional systems were solely based on the dedicated technologies such as Global Positioning System (GPS) and Global Navigation Satellite Systems (GNSS). Recent studies show use of coordinated multi-point techniques in the mmWave wireless access [22,23,24,25], which not only improve coverage and capacity, but also provide more robust communication capabilities. In addition, mmWave technology is also capable of performing accurate user positioning by means of directional beamforming and higher system bandwidth, as shown in [9,10,11,12] with the single BS.

In [22], the author analyzed the coverage performance improvement by serving a user with multiple geographically separated transceivers. Network capacity and coverage gain are also shown in [23], for the heterogeneous systems using stochastic geometry tools. The authors in [24] presented a low complexity cooperation method for the joint transmission, wherein the set of coordinating BSs is obtained by selecting one strongest BS in each tier and extended it in [25] by explicitly considering the impact of blockage density in multi-tier heterogeneous network. Despite the coverage and/or capacity improvements obtained because of multi-point connectivity, as shown in [22,23,24,25], it is yet unclear how concurrent connections with multiple spatially distributed transceivers improve the quality of user experience by providing better positioning quality and achievable user-rate. In this paper, we explore the viability of using coordinated multi-point reception for mmWave based localization and improving the connectivity in the presence of random link blockage. In particular, we focus on time-synchronous conditions, where each user is concurrently communicating with multiple spatially separated receivers. We show that mmWave and massive MIMO with the multi-point connectivity not only contributes in achieving high data rate but also provides more reliable connectivity and accurate positioning capability. The main contribution of this paper are:

- We derive the CRLB for localization in a mmWave based multi-point connectivity scenario, which is subsequently solved to obtain the lower bound on positioning accuracy across spatially distributed receivers.
- We reformulate closed-from expressions for the Fisher Information Matrix (FIM) and provide insight on the dependence of different parameters on the achievable positioning quality.
- We provide a robust receive beamforming strategy that can retain a stable connectivity to combat the uncertainties of mmWave channel.
- We validate the performance of the formulated theoretical bounds via extensive numerical results, i.e., quality of positioning and achievable user-rate with the joint reception.

The reminder of this paper is organized as follows. In Section 2, we provide system architecture and explain the performance metrics. In Section 3, we describe calculation of positioning accuracy using CRLB. The numerical results are presented in Section 4. We give our conclusions in Section 5.

## 2. System Model

We consider uplink in a mmWave based multi-user single-input–multiple-output (MU-SIMO) wireless access, with *K* single antenna transmitters (users) and *B* receivers (Base Stations, BS). The set of all users is denoted as K={1,2,…,K} and B={1,2,…,B} is the set of receiving BSs. Each BS is assumed to be equipped with a uniform-linear-array (ULA) with $N_{R}$ isotropic receive antennas, each antenna with the gain of 0 dBi and the spacing between two adjacent elements is set of λ/2, where λ is the wavelength corresponding to the operating frequency.

**Figure 1:**
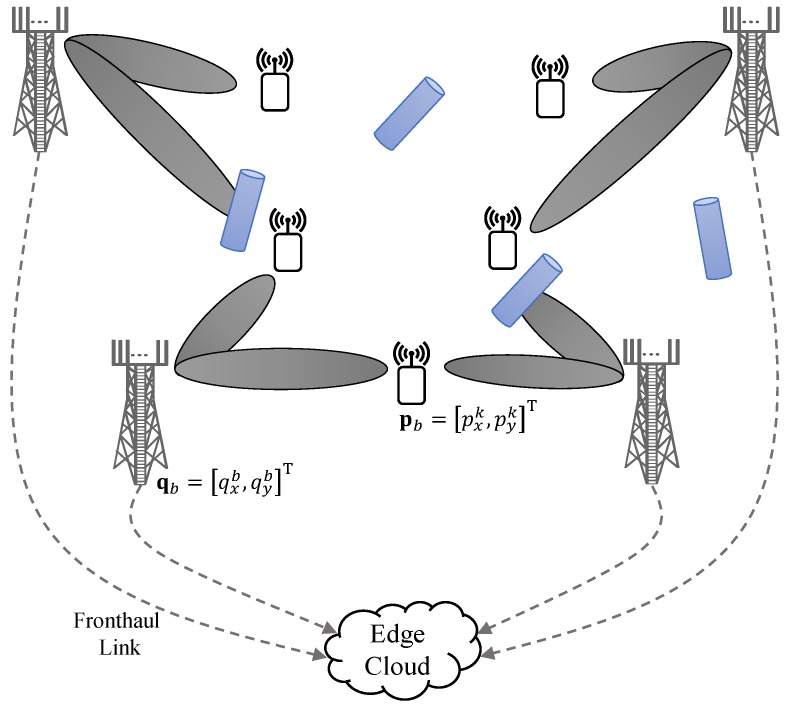
Uplink Communication system model showing receivers (BSs) and transmitters (users) with the LoS link and random blockers.

The problem formulation and proposed method, presented in this paper, are general and can easily be applied to any receive beamforming architecture. However, in this paper, we restrict our analysis to the case when each BS is equipped with an array of antennas and connected to the single baseband processing circuit. However, the analog front-end at each BS supports both antenna specific amplitude scaling and phase-shifting due to antenna specific amplifier and phase-shifter. Thus, one active analog beam in a specific spatial direction can be generated for a given time by appropriate phase and amplitude scaling to each antenna element at each BS. We consider coordinated multi-point reception, whereby all coordinating BSs concurrently receive and coherently combine a time synchronous signal from each active user during baseband signal processing. All BSs are connected to a common baseband-processing-unit (BBU), in the edge cloud by using zero-delay and finite-capacity fronthaul links, as illustrated in Figure 1. BBU performs all the digital signal processing functions, such as resource allocations and joint decoder design for the coordinated reception, over all connected BSs assuming full-CSI of the equivalent channels at the output of analog beamformers (concatenation of analog beamformers and channel vectors).

The two-dimensional (2D) location of *b*th BS is denoted by $q_{b}={\left[q_{x}^{b},q_{y}^{b}\right]}^{T}\;\forall b\in B$ and location of *k*th user is give by $p_{k}={\left[p_{x}^{k},q_{y}^{k}\right]}^{T}\;\forall k\in K$. It is assumed that the location of all BSs $q_{b}\;\forall b$ is fixed and known, while location of all users, $p_{k}\;\forall k$ is not known.

### 2.1. Channel Model

Due to spatially sparse nature of the mmWave channels, because of relatively higher path-loss and low-scattering in comparison to sub-6GHz frequencies, they effectively have “quasi”-optical propagation properties [1], where the line-of-sight (LoS) path is the dominant link that mainly contributes to the communication. For simplicity, we omit the potential NLoS components due to lack of multipath fading, and define the channel between a BS-user pair (b,k) is simply as

(1) $$h_{b,k}=g_{b,k}a_{R}\left(\phi_{b,k}\right)\;,\;\forall b\in B\;,\;\forall k\in K\;,$$

where $g_{b,k}\;=\;h_{b,k}d_{b,k}^{-\vartheta /2}$, in which $h_{b,k}$ represents the complex gain of dominant LoS link with zero-mean and unit-variance, $d_{b,k}$ is the distance between BS-user pair with path-loss factor of ϑ, and $a_{R}\left(\phi_{b,k}\right)\in C^{N_{R}}$ denotes receive antenna array response vector for angle-of-arrival (AoA) $\phi_{b,k}$ with

(2) $${\left[a_{R}\left(\phi_{b,k}\right)\right]}_{m}=e^{j\frac{2\pi d}{\lambda }\left(m-1\right)sin\left(\phi_{b,k}\right)}\;,\;m\in \left\{1,2,\ldots ,N_{R}\right\}\;.$$

It should be noted, because of the higher penetration and path loss [16], mmWave channel between any BS-user pair can be categorized in two states: LoS or blockage. A channel is in blockage state, if the dominant link between a BS-user pair is not available because of some obstacle or resulting path loss is sufficiently high to establish communication. The blockage state is modeled as $h_{b,k}=0^{N_{R}x1},\;\forall b,\;\forall k$. The LoS state is defined in Equation (1), assuming perfect beam-alignment.

### 2.2. Signal Model

**Figure 2:**
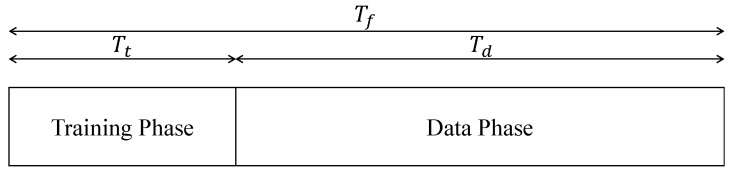
Communication over the frames of duration $T_{f}$, each with a training phase of duration $T_{t}$.

This section provides a generic signal model for system architecture shown in Figure 1. For the simplicity of analysis, we assume that each transmission frame is of duration $T_{f}$ and is divided into two non-overlapping phases: training and data, as illustrated in Figure 2. Training phase of duration $T_{t}$ is reserved for channel estimation and obtaining best receive beams from each BS to each MS. While the UL users are transmitting their pilots for UL training, each BS independently scans through the set of receive beams $w_{b}\in W_{b}$, where $W_{b}$ is the fixed receive beamforming codebook of *b*th BS and each receive beam can be selected through a switching circuitry. Unless stated otherwise, we use superscripts *d* and *p* for the variables related to data and pilot, respectively. The pilot signal for user *k* and BS *b* during the training phase of duration $T_{t}$ for a given receive beam $w_{b}$ can be written as

(3) $$y_{b,k}^{p}\left(t\right)=w_{b}^{H}\left(t\right)h_{b,k}x_{k}^{p}\left(t-\tau_{b,k}\right)+w_{b}^{H}\left(t\right)n_{b}\left(t\right)\;,\;\forall k,\;\forall b\;,$$

where $w_{b}\left(t\right)\in W_{b}$ is the analog receive beamforming vector at time *t* from BS *b*, obtained by both amplitude scaling and phase-shift to each each antenna element. The propagation delay $\tau_{b,k}\;=\;∥q_{b}\;-\;p_{k}∥/c$ is computed for the dominant LoS link with *c* denoting the speed of light, and $n_{b}\left(t\right)\;\forall b$ is circularly symmetric additive-white-gaussian-noise (AWGN) with power-spectral-density of $N_{o}$. Finally, continuous time signal $x_{k}\left(t\right)$ has bandwidth $B_{t}$ and duration *T* with $1/T\int_{0}^{T}\;{|x_{k}\left(t\right)|}^{2}dt\;=\;P_{k},\;\forall k$.

The reminder of duration $T_{d}$ is used for uplink data reception using a fixed analog beam at each BS. For simplicity, we assume full-cooperation scenario, i.e., all BSs receive signal from all active users in the system. However, in practice, the maximum number of coordinating BSs associated to each user is always limited because of path-loss with the distant BSs, finite capacity of fronthaul links and corresponding signaling overhead with the BBU.

### 2.3. Performance Metrics

The system design aims at providing more robust uplink data reception while improving the localization accuracy of each active user using the coordinated multi-point reception. To evaluate that, we have considered two performance metrics: positioning accuracy and effective user-rate, i.e., $Q_{k}$ and $R_{k}\;\forall k$, respectively.

#### 2.3.1. Positioning Accuracy

Positioning accuracy for each user *k* can be determined by the position-error-bound (PEB), computed using the Fisher-information-matrix (FIM) [9]. Assuming the full-cooperation scenario and receive beam $w_{b}\in W_{b}$ of each coordinating BS *b* is used in a time-sequential way. Then, the PEB for *k*th user, $Q_{k}$ is computed as

(4) $$Q_{k}=\sqrt{\sigma_{p_{x}^{k}}^{2}+\sigma_{p_{y}^{k}}^{2}}\;,$$

where $\sigma_{p_{x}^{k}}$ and $\sigma_{p_{y}^{k}}$ are obtained from the inverse of FIM $J_{k}$ for *k*th user [9,12], which includes the Fisher information on the user’s location and corresponding channel coefficients, as given in Equation (28). The construction of FIM matrices $J_{k}\;\forall k$ and detailed derivation for the PEB calculation is provided in Section 3.

#### 2.3.2. Effective Throughput

It should be noted that, as each BS is equipped with a single baseband processing circuit, only one analog beam can be generated in a given direction. Furthermore, the beam remains fixed for the complete data reception phase $T_{d}$. Therefore, aligning the directional beam towards one particular user will results in comparatively poor SNR to all other active users. However, to efficiently utilize the full-cooperation gain, we need to provide comparable SNR to all active users. To do that, we first obtain a compromise receive beam ${\bar{w}}_{b}\;\forall b$ by the superposition of best receive beams of each user and that can obtained by applying appropriate phase-shifts and amplitude scaling to each antenna element, given by

(5) $${\bar{w}}_{b}=\sum_{k}w_{b}^{\left(k\right)}.$$

where $w_{b}^{\left(k\right)}$ denotes the best receive beam for *k*th user from *b*th BS measured during the training phase $T_{t}$. It should be noted that, forming such superposition beam in the analog domain requires both amplitude and phase scaling per antenna element. Therefore, unlike in the plain single beam DFT beamforming, the superposition beams, in general, may not satisfy the uni-modulus constraints on beamforming coefficients. However, uni-modulus constraint can be imposed using the Kronecker decomposition [26], which is left for the future work.

The received signal of *k*th user is given by

(6) $$y_{k}^{d}\left(t\right)=\sum_{b\in B}f_{b,k}{\bar{w}}_{b}^{H}h_{b,k}x_{k}^{d}\left(t-\tau_{b,k}\right)+\sum_{b\in B}\sum_{k^{\prime }\in K\backslash k}f_{b,k}{\bar{w}}_{b}^{H}h_{b,k^{\prime }}x_{k^{\prime }}^{d}\left(t-\tau_{b,k^{\prime }}\right)+\sum_{b\in B}f_{b,k}{\bar{w}}_{b}^{H}n_{b}\left(t\right)\;,$$

(7) $$=f_{k}^{H}{\bar{h}}_{k}x_{k}^{d}\left(t-\tau_{b,k}\right)+\sum_{k^{\prime }\in K\backslash k}f_{k}^{H}{\bar{h}}_{k^{\prime }}x_{k^{\prime }}^{d}\left(t-\tau_{b,k}\right)+f_{k}^{H}\bar{n}\left(t\right)\;,$$

where the effective channel ${\bar{h}}_{k}={\left[{\tilde{h}}_{1,k},{\tilde{h}}_{2,k},\ldots ,{\tilde{h}}_{B,k}\right]}^{T}$, in which ${\tilde{h}}_{b,k}\in C$ is obtained by concatenation of analog beamformer and actual channel. Entries of the effective channel are defined as

(8) $${\tilde{h}}_{b,k}={\bar{w}}_{b}^{H}h_{b,k}\;,\;\forall k,\;\forall b\;.$$

Similarly, $\bar{n}\left(t\right)={\left[{\tilde{n}}_{1}\left(t\right),{\tilde{n}}_{2}\left(t\right),\ldots ,{\tilde{n}}_{B}\left(t\right)\right]}^{T}$, in which ${\tilde{n}}_{b}\left(t\right)={\bar{w}}_{b}^{H}n_{b}\left(t\right)\;,\forall b$. Finally, $f_{k}=\left[f_{1,k},f_{2,k},\ldots ,f_{B,k}\right]$ is user specific digital post-combiner.

Assuming perfect-CSI of the equivalent channel at the output of the analog beamformers, the effective rate for *k*th user, $R_{k}$ is then computed as

(9) $$R_{k}=B_{d}\left(1-\frac{T_{t}}{T_{f}}\right)log_{2}\left(1+\gamma_{k}\left(B\right)\right)\;,$$

where signal-to-interference-plus-noise ratio (SINR) $\gamma_{k}\left(B\right)$ for user k∀k is obtained as

(10) $$\begin{array}{cc} \gamma_{k}\left(B\right) & =\frac{|f_{k}^{H}{\bar{h}}_{k}{|}^{2}P_{k}}{\sum_{k^{\prime }\in K\backslash k}|f_{k}^{H}{\bar{h}}_{k^{\prime }}{|}^{2}P_{k^{\prime }}+{∥f_{k}∥}^{2}\sigma^{2}}\\ & ={\bar{h}}_{k}^{H}{\left(\sigma^{2}I+\sum_{k^{\prime }\in K\backslash k}P_{k^{\prime }}{\bar{h}}_{k^{\prime }}{\bar{h}}_{k^{\prime }}^{H}\right)}^{-1}{\bar{h}}_{k}P_{k}\;, \end{array}$$

assuming linear MMSE receiver, $f_{k}$ is computed as

(11) $$f_{k}={\left(\sigma^{2}I+\sum_{k^{\prime }\in K\backslash k}P_{k^{\prime }}{\bar{h}}_{k^{\prime }}{\bar{h}}_{k^{\prime }}^{H}\right)}^{-1}{\bar{h}}_{k}P_{k}\;,$$

where $P_{k}$ is the maximum uplink transmit power of user *k* and $\sigma^{2}=N_{o}B_{d}$ is the noise power over the signal bandwidth.

In the reference scenario without BS cooperation (or digital post-combiner), each user is only assigned to the closest BS and served with the best matching analog beamformer $w_{b}^{\left(k\right)}$, whereas all other users are treated as interference. In this case, the corresponding SINR is given by

(12) $$\gamma_{k}=\frac{|w_{b}^{{\left(k\right)}^{H}}h_{b,k}{|}^{2}P_{k}}{\sum_{k^{\prime }\in K\backslash k}|w_{b}^{{\left(k\right)}^{H}}h_{b,k^{\prime }}{|}^{2}P_{k^{\prime }}+{∥w_{b}^{\left(k\right)}∥}^{2}\sigma^{2}}\;,\;\forall k,\;\forall b\;.$$

From the above explanation, we can observe the impact of full-cooperation on achievable user-rate and system reliability. For example, use of fully-coordinated multi-point reception will improve system reliability, as all the active users will be connected to all coordinating BSs. However, this will result in lower estimate of the received SINR, because of the compromised receive beam at each BS, which intends to collects signal from all active users rather than focusing the beam towards a specific user at a time. However, the reduced analog beamforming gain can be compensated by efficient joint decoder at the common BBU. Conversely, the non-cooperation scenario, where each BS serves only one closest user for uplink data reception, provides relatively higher estimate of the received SINR because of full analog beamforming gain. However, overall system will be more vulnerable to outage, because of the single point connection.

Clearly, there is tension between achievable analog beamforming gain and reliable connectivity for each user. Our goal here is to understand and quantify this trade-off and provide its affect on the localization accuracy.

## 3. PEB Computation and Channel Estimate

In this section, we elaborate on computation of PEB using CRLB. The CRLB provides a lower bound on the variance of any unbiased estimator of the unknown parameters [27]. In other words, when the CRLB is attained, variance of an estimator should be at least as high as the inverse of Fisher information. For the calculation of PEB, $Q_{k}\;\forall \;k$, we implement a two step approach: First, we obtain FIM for the channel parameters by aggregating the information obtained through scanning all analog receive beams $w_{b}\in W_{b}$ of each coordinating BS b∈B, during the training phase $T_{t}$. Then, we perform bijective transformation to compute these bounds in the position domain and obtain PEB. Finally, we provide channel estimate using the PEB.

### 3.1. FIM: Channel Parameters

Let us define vector of unknown channel parameters as

(13) $$\eta =[\tau^{T},\phi^{T},{g^{R}}^{T},{g^{I}}^{T}]\;,$$

where $\eta \in R^{4BK}$ consist of vectors of delay τ, AoA ϕ, real part of channel coefficients $g^{R}$ and imaginary part of channel coefficients $g^{I}$, such that $\tau \;≜\;[\tau_{1,1},\tau_{1,2},\ldots ,\tau_{B,K}]$, $\phi \;≜\;[\phi_{1,1},\phi_{1,2},\ldots ,\phi_{B,K}]$, $g^{R}\;≜\;\left[g_{1,1}^{R},g_{1,2}^{R},\ldots ,g_{B,K}^{R}\right]$ and $g^{I}\;≜\;\left[g_{1,1}^{I},g_{1,2}^{I},\ldots ,g_{B,K}^{I}\right]$, respectively. Then, the FIM $J_{\eta }\in R^{4BK\times 4BK}$ is structured as

(14) $$J_{\eta }=\left[\begin{array}{cccc} J_{\tau ,\tau } & J_{\tau ,\phi } & J_{\tau ,g^{R}} & J_{\tau ,g^{I}}\\ J_{\phi ,\tau } & J_{\phi ,\phi } & J_{\phi ,g^{R}} & J_{\phi ,g^{I}}\\ J_{g^{R},\tau } & J_{g^{R},\phi } & J_{g^{R},g^{R}} & J_{g^{R},g^{I}}\\ J_{g^{I},\tau } & J_{g^{I},\phi } & J_{g^{I},g^{R}} & J_{g^{I},g^{I}} \end{array}\right].$$

We have adopted orthogonal pilots allocation scheme, where each active user is assigned uncorrelated pilot signals. In addition, each BS independently scans all receive beams and collects respective Fisher information. The receive analog beamforming codebook at each BS consists of DFT steering vectors; further, AoA of each active user can be considered distinct because of random user distribution. Under these assumptions [28], the FIM in Equation (14) can be approximated by the block-diagonal matrices. In particular, we can independently estimate, without any loss in the performance, the unknown parameters of each user with respect of each BS.

For the conciseness of mathematical representation, but without loss of generality, we can reorder the unknown channel parameters in Equation (13) as

(15) $$\eta =[\eta_{1,1},\eta_{1,2},\ldots ,\eta_{B,K}]\;,$$

where $\eta_{b,k}$ represents the channel parameter for user *k* with respect to BS *b*, given by

(16) $$\eta_{b,k}=\left[\tau_{b,k},\phi_{b,k},g_{b,k}^{R},g_{b,k}^{I}\right]\;,$$

and the corresponding FIM $J_{\eta_{b,k}}\in R^{4\times 4}$, portioned into sub-matrices, is given as

(17) $$J_{\eta_{b,k}}=\left[\begin{array}{cccc} J_{\tau_{b,k},\tau_{b,k}} & J_{\tau_{b,k},\phi_{b,k}} & J_{\tau_{b,k},g_{b,k}^{R}} & J_{\tau_{b,k},g_{b,k}^{I}}\\ J_{\phi_{b,k},\tau_{b,k}} & J_{\phi_{b,k},\phi_{b,k}} & J_{\phi_{b,k},g_{b,k}^{R}} & J_{\phi_{b,k},g_{b,k}^{I}}\\ J_{g_{b,k}^{R},\tau_{b,k}} & J_{g_{b,k}^{R},\phi_{b,k}} & J_{g_{b,k}^{R},g_{b,k}^{R}} & J_{g_{b,k}^{R},g_{b,k}^{I}}\\ J_{g_{b,k}^{I},\tau_{b,k}} & J_{g_{b,k}^{I},\phi_{b,k}} & J_{g_{b,k}^{I},g_{b,k}^{R}} & J_{g_{b,k}^{I},g_{b,k}^{I}} \end{array}\right]\;.$$

Each entry in Equation (17) is computed as

(18) $$J_{a,b}≜\frac{1}{\sigma^{2}}\int_{0}^{T}ℜ\left\{\frac{\partial u^{*}\left(t\right)}{\partial a}\frac{\partial u(t)}{\partial b}\right\}dt\;,$$

where u(t) is the noiseless part of the received signal during the training phase, given by

(19) $$u_{b,k}\left(t\right)=w_{b}^{H}\left(t\right)h_{b,k}x_{k}^{p}\left(t-\tau_{b,k}\right)\;,\;\forall k,\;\forall b\;.$$

In the following, we provide main steps to derive first entry in Equation (17) for the brevity. The exact derivations all other entries follow the same procedure, therefore are omitted due to lack of space. Assume that the average symbol power is one and the total transmit power $P_{k}$ with the equal power split per sub-carrier. Then, the OFDM symbol can be defined as

(20) $$x\left(t\right)=\sqrt{\frac{P_{k}}{N}}\sum_{n=1}^{N}e^{j2\pi n\Delta_{f}t}\;,$$

with $\Delta_{f}=1/T$ and *N* is total number of sub-carriers. Using Equation (19), the noiseless received signal $r_{n}$ at *n*th carrier can be written as

(21) $$\begin{array}{ccc} r_{n} & = & \sqrt{\frac{P_{k}}{N}}\sum_{n=1}^{N}w_{b}^{H}h_{b,k}e^{-j2\pi n\Delta_{f}\tau_{b,k}}\\ & = & \sqrt{\frac{P_{k}}{N}}\sum_{n=1}^{N}g_{b,k}w_{b}^{H}a_{R}\left(\phi_{b,k}\right)e^{-j2\pi n\Delta_{f}\tau_{b,k}}\\ & = & \sqrt{\frac{P_{k}}{N}}\sum_{n=1}^{N}g_{b,k}q_{b,k}e^{-j2\pi n\Delta_{f}\tau_{b,k}}\;. \end{array}$$

As given in Equation (18), to derive the expression for the first entry $J_{\tau_{b,k},\tau_{b,k}}$ of the FIM in Equation (17), first we need to compute $\frac{\partial }{\partial \tau_{b,k}}r_{n}$ , given as

(22) $$\frac{\partial }{\partial \tau_{b,k}}r_{n}=\sqrt{\frac{P_{k}}{N}}\left(-j2\pi n\Delta_{f}g_{b,k}q_{b,k}\;e^{-j2\pi n\Delta_{f}\tau_{b,k}}\right)\;.$$

Using Equation (22) in (18), $J_{\tau_{b,k},\tau_{b,k}}$ is computed as

(23a) $$J_{\tau_{b,k},\tau_{b,k}}=\frac{P_{k}^{c}}{\sigma^{2}}|g_{b,k}{|}^{2}{\left(2\pi \Delta_{f}\right)}^{2}n^{2}{\left|q_{b,k}\right|}^{2},$$

following the same steps, all other entries in Equation (17) are computed as

(23b) $$\begin{array}{ccc} J_{\phi_{b,k},\phi_{b,k}} & = & \frac{P_{k}^{c}}{\sigma^{2}}|g_{b,k}{|}^{2}{\left|{\dot{q}}_{b,k}\right|}^{2}\;, \end{array}$$

(23c) $$\begin{array}{ccc} J_{\phi_{b,k},\tau_{b,k}} & = & \frac{P_{k}^{c}}{\sigma^{2}}ℜ\left\{g_{b,k}^{*}q_{b,k}{\dot{q}}_{b,k}^{*}\right\}\;, \end{array}$$

(23d) $$\begin{array}{ccc} J_{\tau_{b,k},\phi_{b,k}} & = & \frac{P_{k}^{c}}{\sigma^{2}}ℜ\left\{jg_{b,k}^{*}q_{b,k}{\dot{q}}_{b,k}^{*}\right\}\;, \end{array}$$

(23e) $$\begin{array}{ccc} J_{g_{b,k}^{R},g_{b,k}^{R}} & = & \frac{P_{k}^{c}}{\sigma^{2}}{\left|q_{b,k}\right|}^{2}\;, \end{array}$$

(23f) $$\begin{array}{ccc} J_{g_{b,k}^{I},g_{b,k}^{I}} & = & \frac{P_{k}^{c}}{\sigma^{2}}{\left|q_{b,k}\right|}^{2}\;, \end{array}$$

where $q_{b,k}\;=\;w_{b}^{H}a_{R}\left(\phi_{b,k}\right)$ and ${\dot{q}}_{b,k}\;=\;a_{R}^{H}\left(\phi_{b,k}\right)D_{b,k}^{H}w_{b}$, in which $D\;\in \;C^{N_{R}xN_{R}}$ is a diagonal matrix with the *l*th entry given by $D\left(l\right)\;=\;j\pi \left(l-1\right)cos\left(\phi_{b,k}\right)$. $P_{k}^{c}=P_{k}/N$ is the power per sub-carrier from user *k*, $\Delta_{f}$ is inter-carrier spacing and *n* denotes the sub-carrier index in the OFDM frame. All the remaining entries in Equation (17) are zero.

### 3.2. CRB for Position

CRLB for the position is computed from the inverse of FIM $J_{{\bar{\eta }}_{b,k}}$ corresponding to positioning parameters ${\bar{\eta }}_{b,k}=\left[p_{x}^{k},p_{y}^{k},g_{b,k}^{R},g_{b,k}^{I}\right]$. To do that, we first obtain position parameters from channel parameters, using the geometric relationship between a BS-user pair [12], as illustrated in Figure 1. For user *k* and BS *b*, parameters $\eta_{b,k}$ and ${\bar{\eta }}_{b,k}$ are related as: $\tau_{b,k}=∥q_{b}-p_{k}∥/c$, $\;cos\left(\pi \;-\;\phi_{b,k}\right)\;=\;\left(p_{x}^{k}\;-\;q_{x}^{b}\right)/c\tau_{b,k}$ and $sin\left(\pi -\phi_{b,k}\right)=\left(p_{y}^{k}-q_{y}^{b}\right)/c\tau_{b,k}$. Let, $T_{b,k}\in R^{4\times 4}$ be the bijective transformation matrix for BS *b* and user *k*, thus $J_{{\bar{\eta }}_{b,k}}$ can be computed as

(24) $$J_{{\bar{\eta }}_{b,k}}=T_{b,k}J_{\eta_{b,k}}T_{b,k}^{H}\;,$$

where

(25) $$T_{b,k}≜\frac{\partial \eta_{b,k}^{T}}{{\partial \bar{\eta }}_{b,k}}\;,\;\forall k,\;\forall b\;,$$

in which

(26) $$T_{b,k}=\left[\begin{array}{cccc} \frac{1}{c}cos\left(\pi -\phi_{b,k}\right) & \frac{1}{c}sin\left(\pi -\phi_{b,k}\right) & 0 & 0\\ -\frac{1}{∥q_{b}-p_{k}∥}sin\left(\pi -\phi_{b,k}\right) & \frac{1}{∥q_{b}-p_{k}∥}cos\left(\pi -\phi_{b,k}\right) & 0 & 0\\ 0 & 0 & 1 & 0\\ 0 & 0 & 0 & 1 \end{array}\right]\;,$$

Finally, the FIM for *k*th user, $J_{k}\in R^{4\times 4}$, which includes the Fisher information on user location and corresponding channel coefficients, is obtained as

(27) $$J_{k}=\sum_{b\in B}\sum_{w_{b}\in W_{b}}J_{{\bar{\eta }}_{b,k}}\left(w_{b,k}\right)\;.$$

and the entries in Equation (4) are calculated as

(28a) $$\begin{array}{c} \sigma_{p_{x}^{k}}^{2}={\left[J_{k}^{-1}\right]}_{1,1}\;, \end{array}$$

(28b) $$\begin{array}{c} \sigma_{p_{y}^{k}}^{2}={\left[J_{k}^{-1}\right]}_{2,2}\;, \end{array}$$

It can be seen from Equation (27) that the positioning accuracy improves with the increase in the number of sounded beams during the beam training phase, as more Fisher information leads to better localization accuracy and, thus, lower PEB. Further, joint reception not only facilitates Fisher information from spatially distributed receivers, but also ensures one or more dominant links to each user, even in presence of random blockers.

### 3.3. Channel Estimation

**Figure 3:**
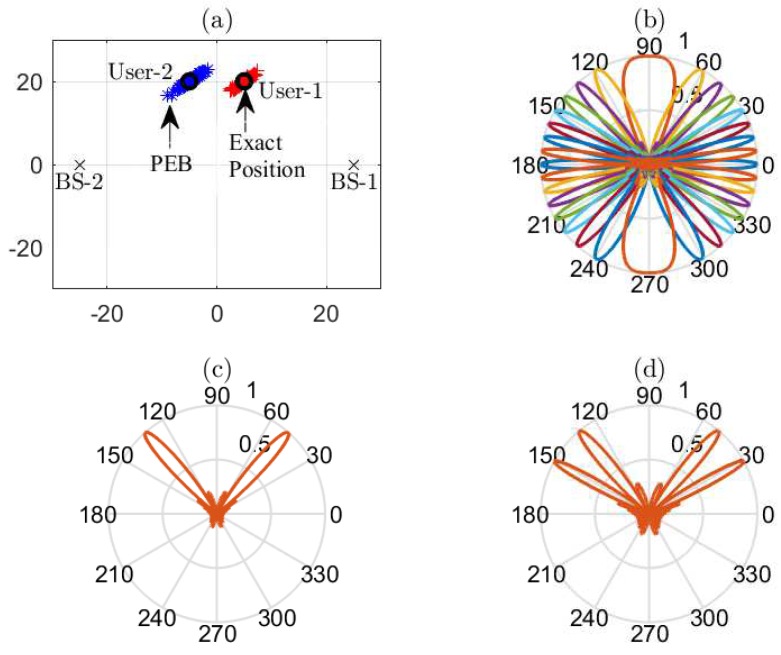
An example system model with: (**a**) communication system with |B|=2,|K|=2 with exact user’s location and corresponding PEB; (**b**) receive beamforming codebook $W_{b}\;\forall b$ with cardinality $|W_{b}|=16$; (**c**) receive beamforming $w_{1}^{\left(1\right)}$ for BS-user pair (1,1) for the non-cooperation scenario; and (**d**) compromise superposition receive beam ${\bar{w}}_{1}$ at BS-1.

The interpretation of the PEB in Equation (28) provides a computationally efficient channel estimate by selecting one point in the PEB region, as shown in Figure 3a. Since the position of each BS $q_{b}\;\forall b$ is fixed and known, therefore, knowing the lower bound on the user’s location and then assuming one possible location within the bound, an estimate of the channel between each BS-user pair (b,k), can be computed as

(29) $${\hat{h}}_{b,k}={\hat{g}}_{b,k}a_{R}\left({\hat{\phi }}_{b,k}\right)\;,\;\forall b\in B\;,\;\forall k\in K\;,$$

where ${\hat{g}}_{b,k}$, is estimated complex gain for the dominant LoS link and $a_{R}\left({\hat{\phi }}_{b,k}\right)$ denotes receive antenna array response vector for estimated AoA ${\hat{\phi }}_{b,k}$ between *b*th BS and *k*th user. Once knowing the estimated user’s location ${\hat{p}}_{k}$, we can easily compute ${\hat{\phi }}_{b,k}$ using $\cos\left(\pi -{\hat{\phi }}_{b,k}\right)\;=\;\left({\hat{p}}_{x}^{k}-q_{x}^{b}\right)/c{\hat{\tau }}_{b,k}$ where ${\hat{\tau }}_{b,k}\;=\;∥q_{b}\;-\;{\hat{p}}_{k}∥/c$. Finally, real and imaginary parts of channel coefficients can be computed directly from ${\left[J_{k}^{-1}\right]}_{3,3}$ and ${\left[J_{k}^{-1}\right]}_{4,4}$, respectively.

## 4. Simulation Results

This section provides numerical results to demonstrate positioning quality and achievable spectral efficiency with respect to different simulation parameters. In particular, we analyze the impact of fully-coordinated multi-point reception on the estimation accuracy and rate performance and compare it with the BS-selection method, where each active user is only assigned to the closest BS. We also evaluate localization accuracy and provide insight on the dependence of different simulation parameters on the achievable positioning quality. We further provide performance in-terms of spectral efficiency and corresponding outage with the compromise receive beam, evaluated over the increasing link-blockage probability.

### 4.1. Simulation Setup

We consider uplink in MU-SIMO wireless communication system with the flat-fading idealized orthogonal frequency-division multiplexing (OFDM) frame, which consists of equally spaced sub-carriers with the inter-carrier spacing of Δf=120 kHz and operating frequency is set to 28 GHz. Furthermore, each frame of duration $T_{f}=10$ ms consists of 1120 symbols, which is aligned with the 5G new-radio (5G NR) numerology [29].

We assume B=2 BSs with $N_{R}=16$ receive antennas. The BSs are placed at opposite corners with the inter-site distance of 50 meters and connected to a common BBU by the fronthaul links with finite capacity and zero delay. Assuming the acquired-CSI from the analog beam sweeping, BBU implements one of two methods: multi-point reception or BS selection. For the multi-point reception method |B|=B, i.e., all coordinating BSs coherently receive uplink data signals with the compromise receive beam ${\bar{w}}_{b}\;\forall b$ from all active users and the corresponding SINR is defined in Equation (10). While in the BS selection method |B|=1, i.e., each user is assigned to the closest BS for uplink data reception with full analog beamforming gain, whereas all other user are treated as interference and the corresponding SINR is defined in Equation (12).

We assume, each user transmits orthogonal pilots in uplink during the training phase, from which, each BS independently scans through the set of receive beams $w_{b}\in W_{b}\;\forall b$. Without loss of generality, we adopt DFT codebook based analog beam training. Then, analog beamforming codebook of BS *b* is with the cardinality of $|W_{b}|=N_{R}\;,\;\forall b$. Each analog beam during the beam training phase is assumed to be estimated from four OFDM pilot symbol, where each symbol is spread over 127 equally spaced sub-carriers (inspired by the synchronization signal block (SSB) numerology in 5G NR [29]). Therefore, with $|W_{b}|=16$, each frame will have a training overhead $T_{t}=64$ symbols, while the reminder of duration is reserved for communication.

Unless stated otherwise, we assume K=2 with the random user distribution, each user with its own channel properties, such as channel gains with the path loss exponent ϑ=2 and AoA with respect to each BS. For simplicity, both users are assumed to have the same maximum uplink transmit power, i.e., $P_{k}=P_{t}\;\forall k$, which is equally divided among all sub-carriers. An example system model is illustrated in Figure 3 for $P_{t}=10$ dBm. All simulation results are averaged over 5000 random channel realizations.

### 4.2. Position Accuracy

**Figure 4:**
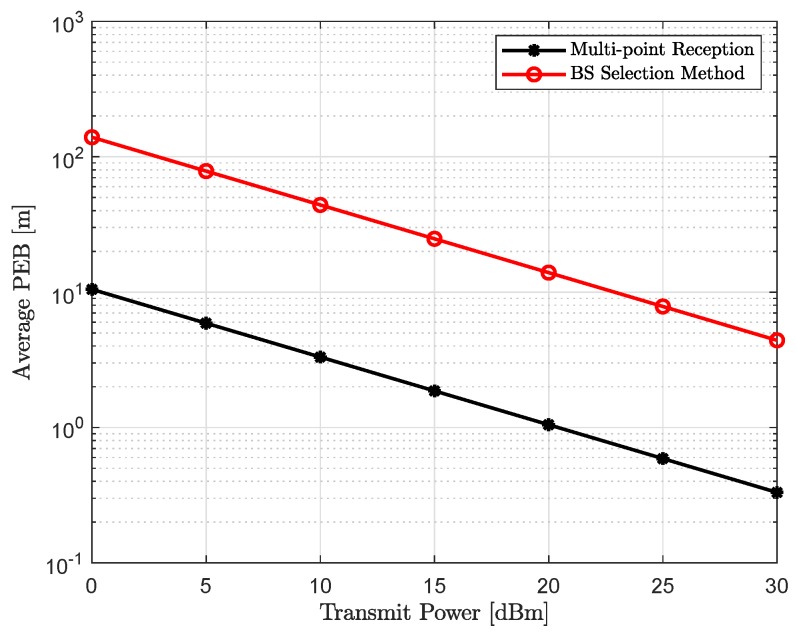
Average positioning accuracy as a function of increasing uplink transmit power $P_{t}$ with the link blocking probability $P_{b}=0$.

**Figure 5:**
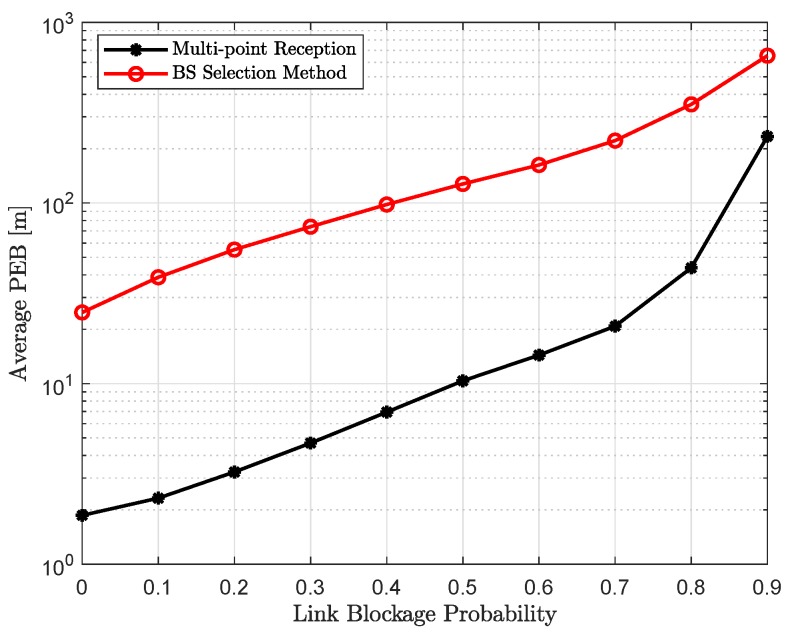
Average positioning accuracy as a function of increasing link blockage probability $P_{b}$ with the uplink transmit power $P_{t}=15$ dBm.

Figure 4 and Figure 5 show the average positioning accuracy with the exhaustive beam training, as a function of increasing uplink transmit power and link blockage probability, respectively. It can be concluded in Figure 4 that positioning accuracy improves with the increase in the uplink transmit power $P_{t}$. Higher the $P_{t}$, better the SNR and that will lead to lower PEB, which can seen from Equation (23). It should be noted that an increase in the number of sounded beams during the beam training phase provides more Fisher information and that leads to better positioning accuracy, and, hence, lower PEB. However, with the increase in link blockage probability $P_{b}$, there will be less active or unblocked beams, resulting in lower Fisher information and higher PEB, as shown in Figure 5, for the increasing link blockage probability.

Performance gap between multi-point reception and BS selection method decreases with the increase in $P_{b}$. The behavior is expected, since, with the higher link blockage probability, there will only be a few active beams that are contributing to the Fisher information.

**Figure 6:**
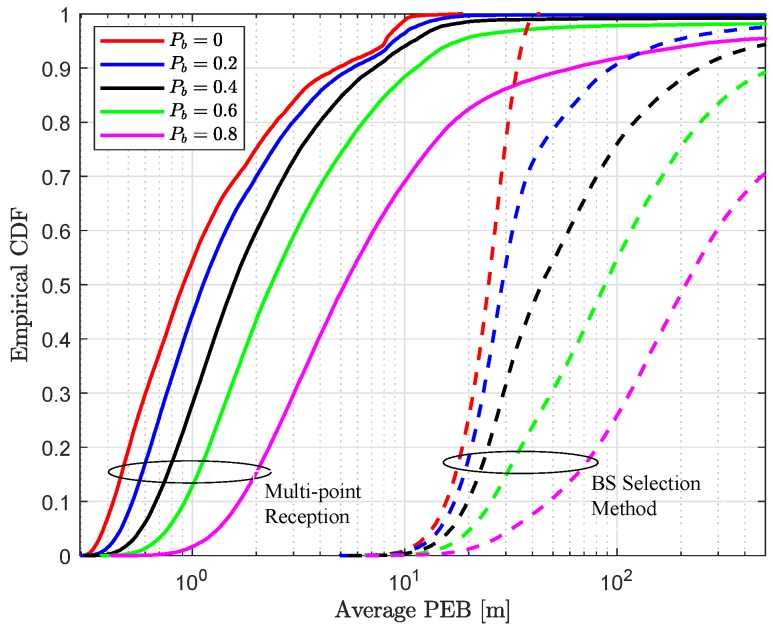
Empirical CDF of average positioning accuracy with different link blocking probabilities $P_{b}$ and uplink transmit power $P_{t}=15$ dBm for multi-point reception (solid line) and BS selection method (dashed line).

Finally, coordinated multi-point reception performs significantly better for all the simulation parameters, i.e., link blockage probability and uplink transmit power. The performance improvement obtained with the joint reception can be clearly seen from the empirical CDF in Figure 6.

### 4.3. Achievable Throughput

**Figure 7:**
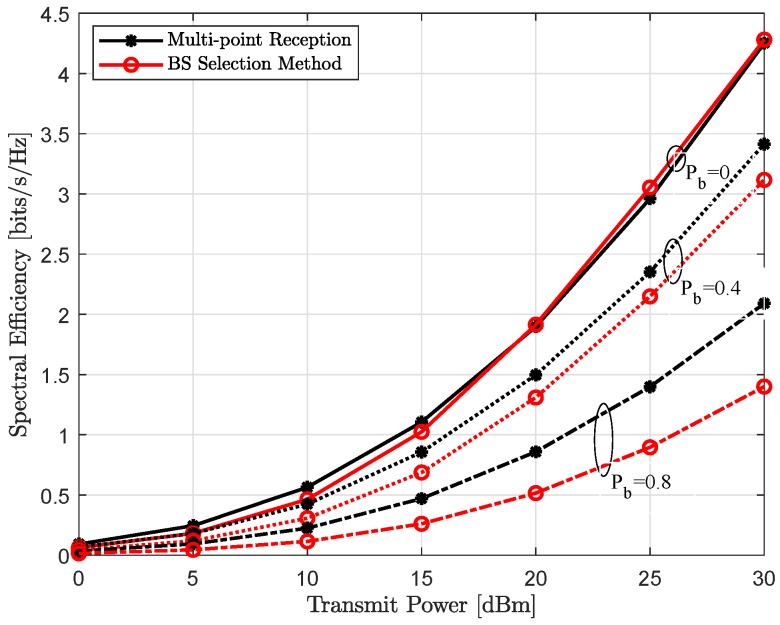
Achievable spectral efficiency assuming exact user location as a function of increasing uplink transmit power $P_{t}$ for the link blocking probability $P_{b}$ of $P_{b}=0$ (solid line), $P_{b}=0.4$ (dotted line) and $P_{b}=0.8$ (dash-dotted line).

**Figure 8:**
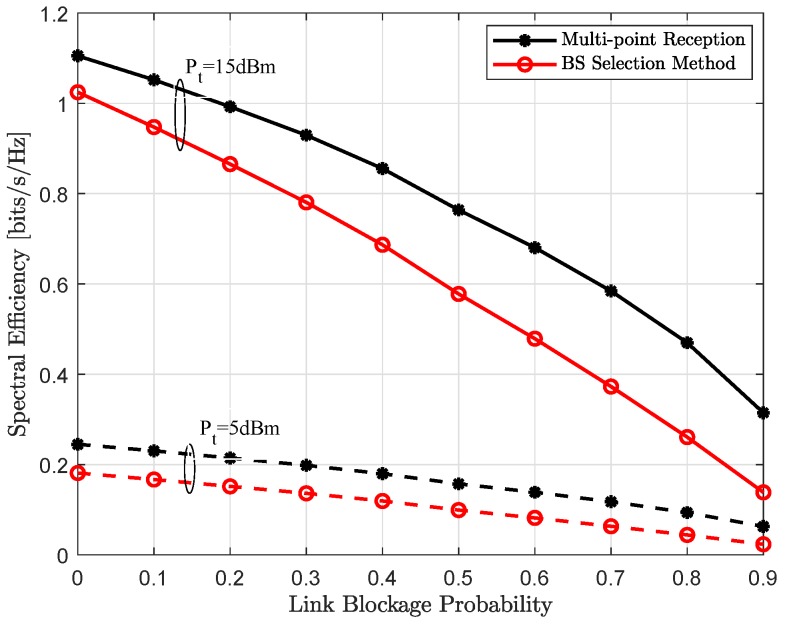
Achievable spectral efficiency assuming exact user location as a function of increasing link blockage probability $P_{b}$ for the uplink transmit power $P_{t}$ of $P_{t}=5$ dBm (dashed line) and $P_{t}=15$ dBm (solid line).

Figure 7 and Figure 8 show the spectral efficiency, assuming exact location of each user is perfectly known, as a function of increasing transmit power and link blockage probability, respectively. As expected, spectral efficiency is monotonically increasing with the increase in uplink transmit power $P_{t}$, shown in the Figure 7. It can be seen, when all LoS links are available, i.e., link blockage probability $P_{b}=0$, both single-BS and multi-point reception provide comparable performance and converges only at very high transmit power. In other words, reduced analog beamforming gain because of the compromise receive beam ${\bar{w}}_{b}\;\forall b$ is recovered with joint reception and efficient interference management to other users. However, for all other cases, coordinated multi-point reception performs significantly better and further this performance gap is also increasing with the increase in link blockage probability $P_{b}$. This behavior is also expected, with the higher $P_{b}$, it is more likely that the dominant LoS link to the connected BS is not available in the single-BS method. However, joint-reception not only provides additional links because of the compromise superposition beam but can also implement efficient interference management to other active users, thus higher user-rate, as shown in Figure 8.

**Figure 9:**
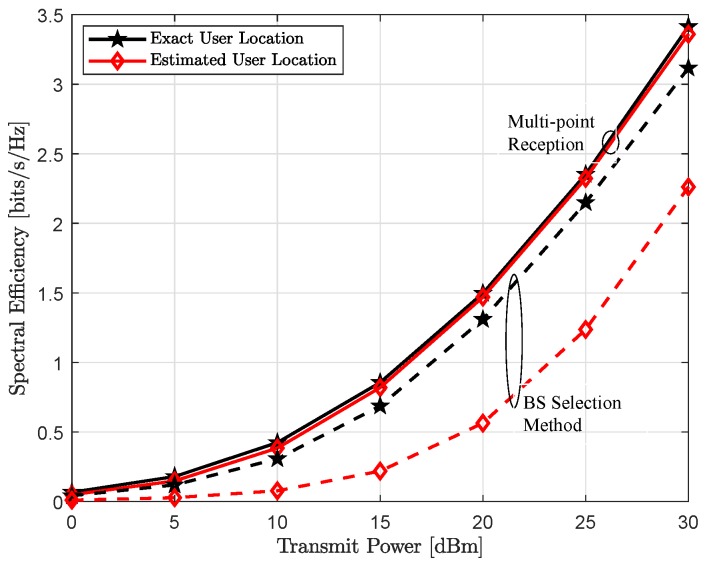
Achievable spectral efficiency with as a function of increasing uplink transmit power $P_{t}$ with the link blocking probability $P_{b}=0.4$ for multi-point reception (solid line) and BS selection method (dashed line).

**Figure 10:**
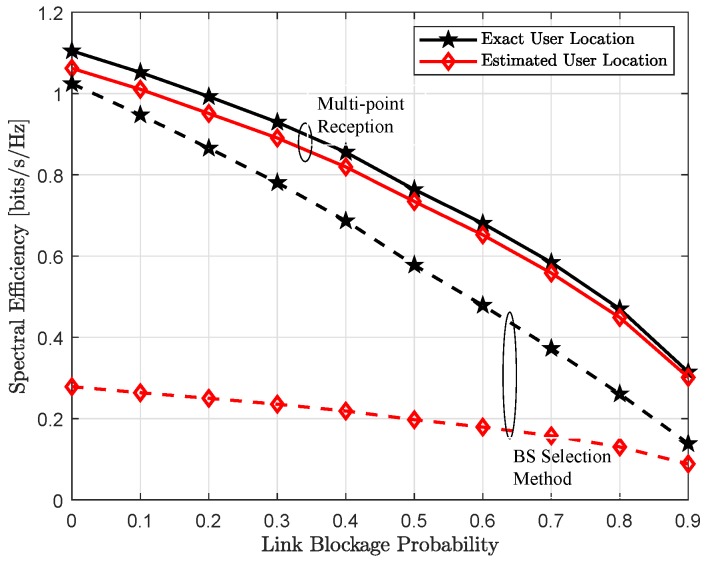
Achievable spectral efficiency as a function of increasing link blockage probability $P_{b}$ with the uplink transmit power $P_{t}=15$ dBm for multi-point reception (solid line) and BS selection method (dashed line).

**Figure 11:**
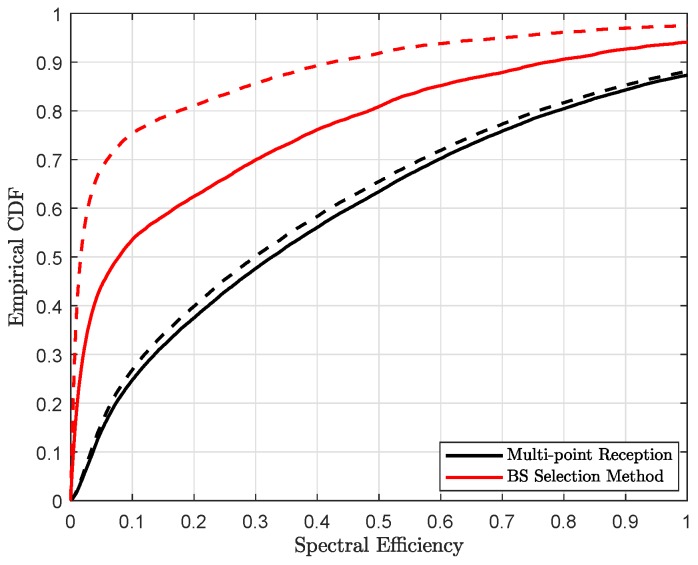
Empirical CDF of achievable spectral efficiency with link blocking probability $P_{b}=0.8$ and uplink transmit power $P_{t}=15$ dBm for exact user location (solid line) and estimated user location (dashed line).

In the Figure 9 and Figure 10, we show achievable spectral efficiency with exact and estimated user’s location, which is related through the analog beam selection $w_{b}^{\left(k\right)}$ between a BS-user pair (b,k). As shown in Figure 4, multi-point reception provides relatively better localization accuracy, thus better estimates of the user’s position with respect to the exact location, which leads to better spectral efficiency, illustrated in Figure 9 and Figure 10. With the coordinated multi-point reception, estimated position accuracy is in the order of sub-meter level. Thus, the difference between exact and estimated position is relatively small. Therefore, we can choose the exact receive beam at each coordinating BS and hence the performance is comparable to the case when user’s location is perfectly known. In contrast, for the BS selection method, the performance gap is because of poor estimate of user location. The performance gain obtained with the joint reception can be clearly seen from the empirical CDF, shown in Figure 11.

## 5. Conclusions

In this paper, we explore the viability of using coordinated multi-point reception to provide better positioning accuracy and higher data-rate in harsh channel conditions. Our proposed approach is scalable to arbitrary mmWave based multi-point configurations and dense deployments. For the evaluation of localization accuracy, we adopted CRLB and provided its dependence of different parameters using the closed-form expressions. It was shown that coordinated multi-point reception provides better positioning quality. By numerical simulations, we verified our analysis and observed that joint reception not only provides high user rate but also stable connectivity and superior localization performance.

For the future work, interest is to consider multi-point sub-set selection and users assignment problem for more reliable and robust joint positioning and communication with fewer BSs.

## References

[1] Rappaport T.S., Sun S., Mayzus R., Zhao H., Azar Y., Wang K., Wong G.N., Schulz J.K., Samimi M., Gutierrez F. (2013). Millimeter Wave Mobile Communications for 5G Cellular: It Will Work!. IEEE Access.

[2] Lemic F., Martin J., Yarp C., Chan D., Handziski V., Brodersen R., Fettweis G., Wolisz A., Wawrzynek J. Localization as a feature of mmWave communication. Proceedings of the 2016 International Wireless Communications and Mobile Computing Conference (IWCMC).

[3] (2018). 3GPP TSG RAN Meeting #80, RP-180897. New SID: Study on NR Positioning. https://portal.3gpp.org/ngppapp/CreateTdoc.aspx?mode=view&contributionId=909020.

[4] El-Sayed H., Athanasiou G., Fischione C. Evaluation of localization methods in millimeter-wave wireless systems. Proceedings of the 2014 IEEE 19th International Workshop on Computer Aided Modeling and Design of Communication Links and Networks (CAMAD).

[5] Kurras M., Thiele L., Peng X., Ishii N. Direction of Arrival Based Positioning in Three Dimensional Coordinates. Proceedings of the WSA 2017 21th International ITG Workshop on Smart Antennas.

[6] Deng H., Sayeed A. Mm-wave MIMO channel modeling and user localization using sparse beamspace signatures. Proceedings of the 2014 IEEE 15th International Workshop on Signal Processing Advances in Wireless Communications (SPAWC).

[7] Andrews J.G., Buzzi S., Choi W., Hanly S.V., Lozano A., Soong A.C.K., Zhang J.C. (2014). What Will 5G Be?. IEEE J. Sel. Areas Commun..

[8] Bielsa G., Palacios J., Loch A., Steinmetzer D., Casari P., Widmer J. Indoor Localization Using Commercial Off-The-Shelf 60 GHz Access Points. Proceedings of the 2018 IEEE Conference on Computer Communications (INFOCOM).

[9] Shahmansoori A., Garcia G.E., Destino G., Seco-Granados G., Wymeersch H. 5G Position and Orientation Estimation through Millimeter Wave MIMO. Proceedings of the 2015 IEEE Globecom Workshops (GC Wkshps).

[10] Shahmansoori A., Garcia G.E., Destino G., Seco-Granados G., Wymeersch H. (2018). Position and Orientation Estimation Through Millimeter-Wave MIMO in 5G Systems. IEEE Trans. Wirel. Commun..

[11] Destino G., Wymeersch H. On the trade-off between positioning and data rate for mm-wave communication. Proceedings of the 2017 IEEE International Conference on Communications Workshops (ICC Workshops).

[12] Kumar D., Saloranta J., Destino G., Tölli A. On Trade-off Between 5G Positioning and mmWave Communication in a Multi-user Scenario. Proceedings of the 2018 8th International Conference on Localization and GNSS (ICL-GNSS).

[13] Saloranta J., Destino G., Wymeersch H. Comparison of different beamtraining strategies from a rate-positioning trade-off perspective. Proceedings of the 2017 European Conference on Networks and Communications (EuCNC).

[14] Saloranta J., Destino G. Reconfiguration of 5G radio interface for positioning and communication. Proceedings of the 2017 25th European Signal Processing Conference (EUSIPCO).

[15] Ghatak G., Koirala R., Domenico A.D., Denis B., Dardari D., Uguen B. Positioning Data-Rate Trade-Off in mm-Wave Small Cells and Service Differentiation for 5G Networks. Proceedings of the 2018 IEEE 87th Vehicular Technology Conference (VTC Spring).

[16] MacCartney G.R., Rappaport T.S., Rangan S. Rapid Fading Due to Human Blockage in Pedestrian Crowds at 5G Millimeter-Wave Frequencies. Proceedings of the GLOBECOM 2017–2017 IEEE Global Communications Conference.

[17] Bennis M., Debbah M., Poor H.V. (2018). Ultra-reliable and low-latency wireless communication: Tail, risk and scale. arXiv.

[18] Tölli A., Codreanu M., Juntti M. Linear Cooperative Multiuser MIMO Transceiver Design with Per BS Power Constraints. Proceedings of the 2007 IEEE International Conference on Communications.

[19] Tölli A., Codreanu M., Juntti M. (2008). Cooperative MIMO-OFDM Cellular System with Soft Handover Between Distributed Base Station Antennas. IEEE Trans. Wirel. Commun..

[20] Tölli A., Pennanen H., Komulainen P. On the Value of Coherent and Coordinated Multi-Cell Transmission. Proceedings of the 2009 IEEE International Conference on Communications Workshops.

[21] Irmer R. (2011). Coordinated multipoint: Concepts, performance, and field trial results. IEEE Commun. Mag..

[22] MacCartney G.R., Rappaport T.S., Ghosh A. Base Station Diversity Propagation Measurements at 73 GHz Millimeter-Wave for 5G Coordinated Multipoint (CoMP) Analysis. Proceedings of the 2017 IEEE Globecom Workshops (GC Wkshps).

[23] Maamari D., Devroye N., Tuninetti D. (2016). Coverage in mmWave Cellular Networks With Base Station Co-Operation. IEEE Trans. Wirel. Commun..

[24] Skouroumounis C., Psomas C., Krikidis I. Low-Complexity Base Station Cooperation for mmWave Heterogeneous Cellular Networks. Proceedings of the 2016 IEEE Global Communications Conference (GLOBECOM).

[25] Skouroumounis C., Psomas C., Krikidis I. Low complexity base station cooperation in cellular networks with blockages. Proceedings of the 2016 IEEE Wireless Communications and Networking Conference.

[26] Zhu G., Huang K., Lau V.K.N., Xia B., Li X., Zhang S. (2017). Hybrid Beamforming via the Kronecker Decomposition for the Millimeter-Wave Massive MIMO Systems. IEEE J. Sel. Areas Commun..

[27] Kay S.M. (1993). Estimation theory. Fundamentals of Statistical Signal Processing.

[28] Abu-Shaban Z., Zhou X., Abhayapala T., Seco-Granados G., Wymeersch H. (2018). Error Bounds for Uplink and Downlink 3D Localization in 5G Millimeter Wave Systems. IEEE Trans. Wirel. Commun..

[29] (2018). 3GPP TSG RAN Meeting #82, TS 38.300. NR and NG-RAN Overall Description. https://portal.3gpp.org/desktopmodules/Specifications/SpecificationDetails.aspx?specificationId=3191.

